# Developing Suitable Buffers to Capture Transport Cycling Behavior

**DOI:** 10.3389/fpubh.2014.00061

**Published:** 2014-06-05

**Authors:** Thomas Madsen, Jasper Schipperijn, Lars Breum Christiansen, Thomas Sick Nielsen, Jens Troelsen

**Affiliations:** ^1^Department of Sport Science and Clinical Biomechanics, University of Southern Denmark, Odense, Denmark; ^2^Department of Transport, Transport Policy and Behaviour, DTU Transport, Lyngby, Denmark

**Keywords:** cycling, transport, GPS, built environment, physical activity, MAUP, buffers

## Abstract

The association between neighborhood built environment and cycling has received considerable attention in health literature over the last two decades, but different neighborhood definitions have been used and it is unclear which one is most appropriate. Administrative or fixed residential spatial units (e.g., home-buffer-based neighborhoods) are not necessarily representative for environmental exposure. An increased understanding of appropriate neighborhoods is needed. GPS cycling tracks from 78 participants for 7 days form the basis for the development and testing of different neighborhood buffers for transport cycling. The percentage of GPS points per square meter was used as indicator of the effectiveness of a series of different buffer types, including home-based network buffers, shortest route to city center buffers, and city center-directed ellipse-shaped buffers. The results show that GPS tracks can help us understand where people go and stay during the day, which can help us link built environment with cycling. Analysis showed that the further people live from the city center, the more elongated are their GPS tracks, and the better an ellipse-shaped directional buffer captured transport cycling behavior. In conclusion, we argue that in order to be able to link built environment factors with different forms of physical activity, we must study the most likely area people use. In this particular study, to capture transport cycling, with its relatively large radius of action, city center-directed ellipse-shaped buffers yielded better results than traditional home-based network buffer types. The ellipse-shaped buffer types could therefore be considered an alternative to more traditional buffers or administrative units in future studies of transport cycling behavior.

## Introduction

Built environment characteristics can influence health; both directly and indirectly, linked to health-related behavior and activities in general (1–4). Notwithstanding, ongoing discussions on defining the relevant geographic extent when studying built environment characteristics have not yet resulted in a commonly accepted “best practice” for defining neighborhoods. Different ecological and multilevel analyses often use varying notions of neighborhood which has shown to be problematic (5, 6). The modifiable areal unit problem (MAUP) is often discussed as it is related to the geographic scale and unit of aggregation. Correlation and association might change unpredictably as the scale and unit of aggregation changes (7). This is challenging as “any study about neighborhoods is a spatial investigation” (8) and “effective neighborhoods, such that they exist as contiguous geographic areas, are not likely to be neat circles” (7). Furthermore, Kwan has argued that the uncertain geographic context problem (UGCoP) is as fundamental as MAUP as the spatial and temporal uncertainty of where, when, and how long individuals experience environmental influences is great (9). This might explain inconsistencies in research findings as the commonly used methods may not correctly represent the spatial area in which the behavior in question occurs (5, 10, 11). Administrative units, which are often used as “neighborhoods,” simplify and fragment space which leads to potential misestimating of associations between the built environment and behavior (5, 12). Nevertheless, in real life it might be necessary to simplify assumptions to make a “draft of reality” that allows us to conceptualize neighborhoods in a useful way (7). Simplification and the fact that information is often easily available for administrative units might explain the numerous studies using them. Recommendations on using person-centered neighborhoods to better reflect a more reasonable exposure area have been published, and these neighborhoods are typically described as centers (e.g., home address) with boundaries created on the basis of a threshold distance. This threshold distance varies from study to study, but should ideally be related to the outcomes of interest, contextual factors, and study area (5).

Built environment correlates of walking have been reported (13–15), but literature concerning the relationship between the built environment and transport cycling is still limited. As many major cities and countries have discovered the potential of bicycles to replace cars on shorter trips in everyday transport, it is relevant to study the correlates of transport cycling (16). In Denmark, cycling holds an important place in everyday life with a cycle mode share of 16% of all trips (17, 18). More knowledge on how the built environment is associated with transport cycling is still wanted to be able to further increase the cycle mode share. Studies have shown that several factors are important for cycling, e.g., distance, network layout/street connectivity, residential density, land use mix, bicycle infrastructure, continuity of cycle lanes, traffic-controlling systems (19). The geographic area in which factors should be measured is not clear though. A geospatial analysis should use appropriate buffers, instead of fixed administrative areas (census tracts, zones), circular buffers, or even whole cities, yet there is limited empirical data to support an informed choice of study area (1, 5, 7, 20). It seems necessary to study cyclist’s behavior and construct more appropriate buffers in relation to size and shape to better capture the environment cyclists’ transport behavior occurs in (6, 20). The interaction with the built environment often results in asymmetric and directional behavior which varies accordingly to destinations of interest (sports, work, education, recreational areas, retail, etc.). Many cyclists can easily cover 5 km, in approximately 20 min (21), but they will most likely do so in a certain area and, for many transport purposes, probably in the direction a cluster of daily destinations. When studying active transport and human movement in general, people only access a fraction of the buffer areas commonly used for analysis (11, 22). The spatial uncertainty challenges might be lessened if the spatial unit is defined on the basis of behavior and contextual environment (9, 23).

Numerous papers have addressed the challenge of creating suitable buffers for different types of behaviors and discuss the use of, e.g., activity spaces, home ranges, kernel density estimations, daily life centers (hotspots), road network buffers, relative time travel zones, or similar methods (1, 10, 24–26). Rainham et al. emphasized the need for better knowledge of the dynamics of human movement and discuss the issues of spatial bounding, for example by using advanced data collection methods such as GPS technology. The aim should be to collect and analyze space–time–activity data where locations and movement of individuals can be followed and visualized as continuous tracks (1). Cycling behavior can be studied by GPS and provide empirical data to construct buffers which better capture cycling activity and allow for detection of destinations.

As Spielman and Yoo put it: “If you are going to spend time and money painting a picture of the relationship between the environment and health invest in the frame – unless the frame is well-designed, the painting is not going to be very good” (7).

Perchoux et al. outline components of mobility in relation to activities which are “daily life centers” (home, work, etc.), “clusters of minor activities locations” (restaurants, banks, daily shopping, etc.), “circulation corridors” (the familiar routes between usual places), and “transport interfaces” (underground stations or car parks) (5). In the present study, we used home as starting point, the city center as activity location cluster, and the shortest route network from home to city center as corridor.

The purpose of the present study is to develop a method using GPS technology and geographical information systems to analyze behavioral patterns and construct buffers that can be used for analyses of the relation between built environment and transport cycling. The percentage of GPS points per square kilometer is used as indicator for buffer effectiveness, attempting to reduce the non-frequented area of the buffer and address both MAUP and UGCoP (9). We hypothesize that the method is viable and that in the case of transport cycling, the further people live from a cluster of daily destinations, i.e., a city center, the more elongated and city center-directed their buffer should be in order to effectively capture transport cycling behavior.

## Materials and Methods

### Participants, city layout, and design

The participants were recruited among the regular cyclists (*N* = 331) who participated in the Danish part of the IPEN study (*N* = 642)^1^ conducted in Aarhus. Aarhus is the second largest city in Denmark (323.893 inhabitants and approximately 470 km^2^) and has a cycle mode share of approximately 17% of all trips (27). Aarhus is a typical Danish fjord city with a waterfront and relatively large differences in altitude from the inner city to the city outskirts. The city layout is traditional and consists of ring roads and main roads leading from the suburbs toward the inner city with smaller crossroads. There is a well-connected cycle path network throughout the city and in general good cycle facilities compared to many other European and American cities. Furthermore, Aarhus is an educational hub and approximately 50,000 students live and study in Aarhus. This leads to a relatively high cycling mode share (17%) as Danish students traditionally cycle more than other population groups (17). Table 1 shows the characteristics of the participants.

**Table 1:** **Participants’ characteristics**.

Participants’ characteristics	Female	Male	Total
Participants, no. (%)	51 (65.4)	27 (34.6)	78
Age (years, mean ± SD)	34.7 ± 14.0	43 ± 12.1	37.5 ± 13.9
<30 years (%)	25 (49.0)	4 (14.8)	29 (37.2)
30–40 years (%)	11 (21.6)	8 (29.6)	19 (24.4)
40–50 years (%)	3 (5.9)	5 (18.5)	8 (10.3)
50–60 years (%)	7 (13.7)	7 (25.9)	14 (17.9)
Over 60 years (%)	5 (9.8)	3 (11.1)	8 (10.3)
Education and employment status, no. (%)			
Municipal primary and lower secondary school	1 (1.9)	1 (3.7)	2 (2.6)
Vocational	3 (5.9)	7 (25.9)	10 (12.8)
Upper secondary/high school	15 (27.4)	0 (0.0)	15 (19.2)
Higher education	32 (62.8)	19 (70.4)	51 (65.4)
Working	24 (47.1)	20 (74.1)	44 (56.4)
Studying	22 (43.14)	4 (14.8)	26 (33.3)
Welfare, pension	5 (9.8)	3 (11.1)	8 (10.3)

The 331 participants who stated in the IPEN questionnaire that they were regular cyclists were invited to participate in the GPS study. Ninety-three joined the study, and 78 met the inclusion criteria of having at least one valid GPS-measured cycle trip during the study period.

### GPS tracking

Participants were asked to wear the GPS (QStarz BT-Q1000X Travel Recorder; 15 s sampling interval) for 7 days (Wednesday to Wednesday) to be able to detect differences in travel behavior between weekdays and weekends. The QStarz BT-Q1000X Travel Recorder has shown to be an accurate GPS receiver with long battery life well-suited for free-living studies (28, 29). The cyclists were instructed to wear the GPS for transport cycle trips only, as other modes of transport were not of interest in the present study. Cycling for transport includes cycling to, e.g., work, education, shopping, sport facilities, etc. and does not include recreational trips. Non-transport trips were excluded based on the trip description in the diaries. One potential challenge with the use of GPS is the classification of transport modes after data collection (30–32) and by instructing the participants to limit the use to transport cycling only we hoped to overcome this. Daily SMS text messages were sent in the morning to remind participants to bring the GPS device and in the evening to remind them to charge the device if necessary. GPS device configuration and data download were performed using the open source BT747 GPS data logger software^2^.

### GPS data processing

GPS trackers yield massive data quantities and in order to make the best of the data, we decided to process and clean the data using the personal activity location measurement system (PALMS) which is developed and maintained by the University of California, San Diego. PALMS uses extreme differences in speed and altitude to filter out “bad” GPS point, and produces data sets that, among other, include trips separated by trip mode (30). The results from PALMS were imported into geographical information system software (ArcGIS 10.1) for further analysis.

Even though we only intended to collect GPS data for cycling trips, there were a large number of static GPS points in our dataset. Random manual inspection of the data revealed that this was primarily due to participants forgetting to turn the GPS device off at home or at their destination. In order to create cyclist buffers, more than three million GPS points were reduced to approximately 70,000 by excluding all stationary points and only using the points that PALMS had classified as being part of a trip. The GPS track points make it possible to outline the true geographic extent of transport cycling for each participant during the study period.

### Creation of buffers

From the GPS trip points, we calculated standard deviational (SD) ellipse buffers which are widely recognized as a good summary of the spatial patterns derived from all data collected with the GPS device (1, 26). We used both 1 and 2 SD ellipse buffers to be able to analyze the difference in area and effectiveness. The 1 SD ellipses theoretically include 68% of all the GPS points, whereas the 2 SD ellipses contain 95% of the GPS points. Furthermore, 1- and 2-km network buffers were constructed around every participant’s residential address.

On the basis of the hypothesis that much cycling for transport would be directed toward a cluster of destinations, the location with the highest concentration of daily destinations was calculated for Aarhus, which is a city with a strong center orientation. Based on their relevance as regularly reoccurring destinations, the following building categories were counted as daily destinations: retail, supermarkets, sport-clubs, schools and educational institutions, and cultural facilities such as libraries and theaters. The centroid of the location with the highest density was used as city center point.

Based on the location of the highest concentration of daily destinations (the city center), we developed different types of directional buffers. Shortest route buffers (500, 750, and 1000 m wide respectively) from home to the city center were created as well as ellipse-shaped buffers based on the Euclidian distance (as the crow flies) and bearing (direction) from home to the city center. We created three ellipse-shaped buffers with a fixed width of 500, 750, and 1000 m, respectively, and a length based on the distance from home to city center, to which an additional 500 m were added (250 m in each end of the ellipses). Finally, we created one buffer with a variable width based on the distance to the city center; respondents living closer than 2 km from the city center were assigned a buffer with a 1-km width, whereas respondents living more than 5 km from the city center were assigned a buffer with a 500-m width. The buffer width for respondents living between 2 and 5 km from the city center gradually decreased from 1 km to 500 m. The aim was to decrease buffer size but still capture as many GPS points as possible in order to create buffers that most effectively capture transport cycling behavior without including large areas where people never cycle.

### Statistics and calculations

Descriptive statistics (age, education, gender, number of cycling trips, cycled kilometers, and average trip length) were calculated for all participants, and all GPS cycle tracks were plotted on a map. Per person, we calculated the shortest network distance between home and all GPS points. Based on this, we calculated the distance within which a certain percentage of GPS points were located (1, 5, 10, 25, 50, 75, 90, and 100%). Pearson’s correlation coefficients between distance from home address to city center and ellipse circumference, ellipse area, and ellipse length–width ratio were also calculated.

For each buffer type, we calculated the buffer area in square kilometers, the number of GPS points, and the percentage of GPS points per square kilometer. To be able to test which buffer performs best in capturing transport cycling behavior, we analyzed the “effectiveness” of different buffer types by comparing the relative density of GPS points. We hypothesized that buffers with a higher relative density of GPS points were more effective at capturing cycling behavior. Regression analyses were conducted to test buffer shape and effectiveness where buffer types without significant differences in effectiveness between respondents were considered more appropriate. Not finding a difference between participants indicates that the buffers are equally good at capturing respondents’ GPS points, regardless of how far from the city center they live.

All statistical analysis were performed in STATA version 11 (STATA Corp., Fort Walton, TX, USA) and an alpha level of 0.05. Data conversions between ArcGIS and STATA, and vice versa, were carried out using Stat/Transfer, Circle Systems.

## Results

Table 1 shows the characteristics of the participants regarding age, gender, educational level, and employment status. Table 2 shows the transport cycling in detail for the 7 days, with almost 25 cycling trips per person on average, and an average trip length of just under 500 m.

**Table 2:** **Transport cycling for 7 days**.

	Female	Male	Total
Cycling trips	22.6 ± 10.9	28.2 ± 25.4	24.5 ± 17.4
Cycling kilometers	9.8 ± 11.9	15.2 ± 21.0	11.7 ± 15.8
Average trip length (m)	484.1 ± 924.0	494.5 ± 418.3	487.7 ± 783.3

Visual inspection of the GPS tracks and analysis of GPS point distances showed patterns in transport cycling behavior that confirmed the initial hypothesis. Fifty percentage of GPS points were located within 1440.9 m for people living within 2 km of the city center. For people living more than 2 km from the city center, the distance to capture 50% of all GPS points was 2548.2 m. The maps and distance analysis indicate that people living further away from the city center have a transport pattern between home and city center whereas people living closer to the city center had points spread more equally in various directions.

**Figure 1:**
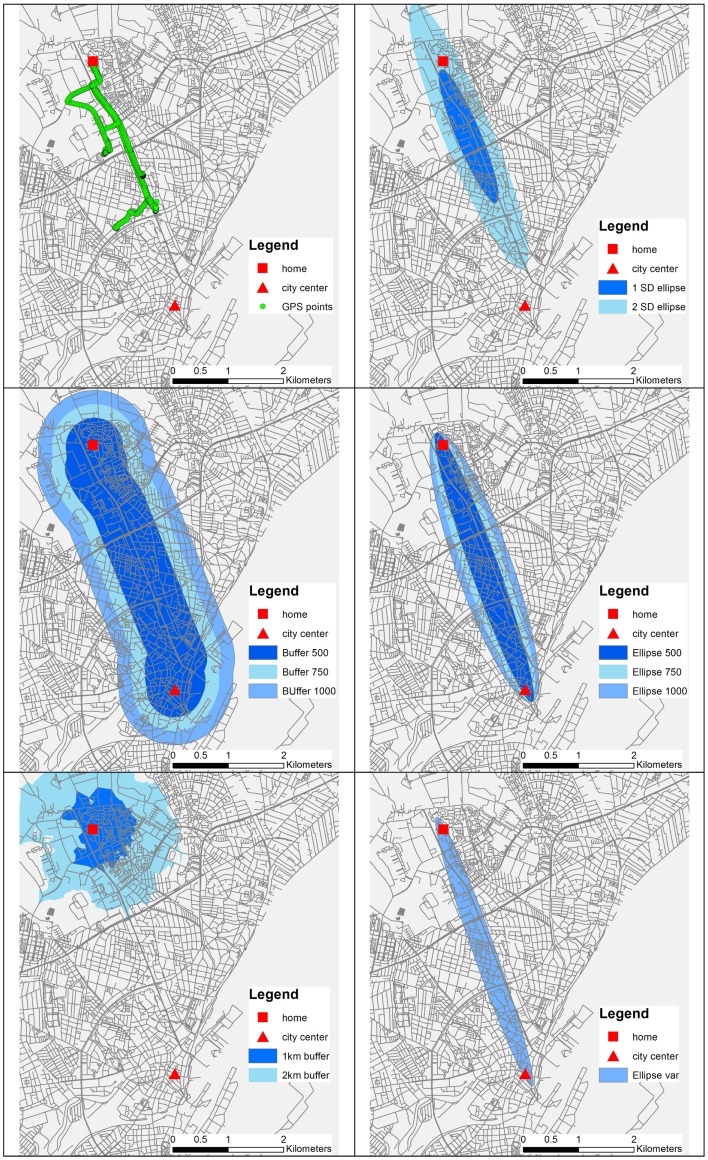
**The six buffer types and sub-buffers for one participant**. The figure displays how some buffers are developed on the basis of the GPS track and directed toward the city center, while the more traditional buffers are created solely on home address and information derived via the geographical information system.

This supports that the transport pattern differs according to where people live in the city, not only regarding their closest neighborhood, but also related to the distance from the city center, which should be taken into account when constructing transport cycling buffers. The buffer types with different distance thresholds are depicted in Figure 1.

The results from the analyses are shown in Table 3. For every buffer, the area, number of GPS points, percentage of GPS points, density (GPS points per square kilometer), and relative density (percentage GPS points per square kilometer) were calculated.

**Table 3:** **Buffer area, GPS points, percentage GPS points, density (GPS points per square kilometer), and relative density (percentage GPS points per square kilometer)**.

Buffer characteristics (mean ± SD)	Area in km^2^	GPS points	% GPS points	Density, GPS points/km^2^	Relative density, % GPS points/km^2^
1 SD ellipse	6.84 (0.9)	594.2 (503.2)	64.5 (7.6)	225.7 (232.4)	43.0 (70.7)
2 SD ellipse	27.3 (3.5)	874.3 (631.3)	97.9 (2.3)	84.6 (84.9)	16.3 (26.4)
1-km network buffer	1.57 (0.2)	269.6 (277.1)	33.4 (20.3)	171.5 (167.1)	21.4 (13.2)
2-km network buffer	6.75 (0.8)	443.9 (321.4)	56.4 (25.6)	65.8 (46.4)	8.3 (3.5)
Shortest route buffer (500 m)	4.55 (3.5)	410.9 (362.6)	50.4 (27.2)	124.9 (137.6)	15.6 (11.1)
Shortest route buffer (750 m)	7.4 (5.2)	482.6 (380.7)	59.4 (27.5)	86.7 (82.9)	10.9 (7.2)
Shortest route buffer (1000 m)	10.6 (6.9)	533.0 (394.3)	65.1 (26.8)	64.1 (56.4)	7.9 (4.7)
Ellipse (500 m)	1.57 (1.3)	232.2 (290.9)	28.6 (21.8)	237.1 (377.4)	29.3 (28.9)
Ellipse (750 m)	2.36 (1.9)	288.8 (326.4)	35.0 (23.7)	190.0 (263.7)	23.3 (20.4)
Ellipse (1000 m)	3.14 (2.5)	328.1 (338.2)	40.0 (24.9)	159.2 (202.7)	19.6 (16.4)
Variable buffer	1.62 (0.8)	250.1 (295.2)	30.1 (21.9)	186.4 (234.8)	23.03 (18.4)

In theory, 1 and 2 SD ellipses capture 68 and 95% of all GPS points. But Table 3 shows that the results for the 1 and 2 SD buffers were 64.5 and 97.8%, respectively. These discrepancies are due to the elliptic form making it impossible to include the exact percentage of GPS points. As a benchmark value, the 1 SD buffer captures 43% of GPS points per square kilometer, but as SD buffers can only be constructed when GPS data is available, they are not suitable for studies without GPS data. The most effective constructed buffer type is the directional ellipse 500 as it captures 29.3% of GPS points per square kilometer. The ellipse-shaped buffers capture a relative high percentage of GPS points while covering a smaller area with direction toward the city center.

The association between distance from home address to city center and percentage of GPS points captured by the network buffers showed negative correlation coefficients of −0.23 (*p* < 0.05) for the 1-km network buffer and −0.46 (*p* < 0.05) for the 2-km network buffer. The correlation coefficients between distance from home address to city center and ellipse circumference, ellipse area, and ellipse length–width ratio were also calculated. The coefficients were 0.40 (*p* < 0.05), 0.23 (*p* < 0.05), and 0.33 (*p* < 0.05), respectively. The coefficient between distance from home address to city center and total GPS points was −0.0015 (*p* = 0.98) (data not shown). The above mentioned coefficients indicate that people living further away from the city center had larger and more stretched ellipses but no difference in total number of GPS points collected during cycling trips.

Table 4 presents the results from the regression analyses between the percentage of GPS points inside the 11 buffer types and distance from home to center. The results are supported by additional regression analyses between length–width ratio and distance from home address to city center. The coefficient for 1 and 2 SD was 0.39 (*p* < 0.005), for 1- and 2-km network buffers −0.01 (*p* < 0.05) and −0.03 (*p* < 0.001), respectively (data not shown). The further away people live from the city center, the lower the percentage of GPS points 1- and 2-km network buffers capture, even though the coefficients are small they are significant. As opposed to this, none of the elliptical buffers had significant coefficients (*p*-values between 0.096 and 0.784) indicating a more constant capacity to capture GPS points regardless of the distance from home to city center. The 500, 750, and 1000 m shortest route buffers had coefficients of 0.02 (*p* < 0.005), 0.02 (*p* = 0.05), and 0.01 (*p* = 0.1), respectively.

**Table 4:** **Results for the regression analysis between the percentage of GPS points inside the 11 buffer types and distance from home to center**.

Buffer type	Coefficient	*p*-Value
1 SD ellipse	−0.007	0.002
2 SD ellipse	0.001	0.052
1-km network buffer	−0.01	0.048
2-km network buffer	−0.03	0.001
Shortest route buffer (500 m)	0.02	0.008
Shortest route buffer (750 m)	0.02	0.050
Shortest route buffer (1000 m)	0.01	0.108
Ellipse (500 m)	−0.002	0.783
Ellipse (750 m)	0.002	0.784
Ellipse (1000 m)	0.006	0.452
Variable buffer	−0.01	0.096

## Discussion

This study aimed to combine a mixture of hypothesized reasoning and exploratory analysis to develop methods to determine buffering-radius size and buffer shape, as recommended by Chaix et al. (24). The methods can be used in studies of active transport behavior or other types of behavior where researchers are interested in knowing where study participants primarily go and stay in order to get a more precise comprehension of the concept “neighborhood.” In line with Boruff et al. (10), we distinguished and tested a variety of buffers that can be used when studying the relationship between the built environment and active transport. People living within the city center have easy access to a variety of destinations which means that a circular or network buffer will capture most of their activity while this is not the case for people living further away from a city center. Based on a comparison of the effectiveness of 11 different buffer types, we argue for different neighborhood buffer types, shapes, and sizes to mimic the behavior in question. Using ellipse-shaped buffers directed from the residential address to a city center (i.e., a high concentration of daily destinations) is, to our knowledge, a novel way of delineating an active travel neighborhood.

We assume that people in general transport themselves toward meaningful destinations in a rather direct way and therefore have confidence that the methodology holds for cities with one or more areas with a high concentration of daily destinations. However, it is unlikely that the methodology holds for city layouts without clear concentrations of daily destinations. Similar analyses of cycling in other types of cities might help overcome this challenge, and other types of buffers could be constructed to reflect cycling behavior.

There is an absence of studies that provide measures of “true” exposure to environmental factors even though the discussion on buffer types has been around for years and several studies conclude that a standard network buffer around the home might not adequately reflect the activity space examined in the studies (5, 11). Villanueva et al. (11) show how children only access up to a quarter of the calculated traditional “neighborhood” (defined with an 800- or 1600-m network buffer) and thus not travel completely within or use all of their neighborhood area. Clustered destinations and specific directions could be reasons for this and future studies should explore the directional movement and spatial orientation of visited (or probable) destinations to explore the built environment characteristics people are exposed to (11).

The question is whether we want “neighborhood” buffers to capture most of the expected environmental “exposure” as well as include large areas that people never visit. Or do we measure the “possible exposure” less accurate by not including large areas of non-exposure? Using GPS-derived activity space is often not possible in large population studies (1) but the use of GPS-derived buffer construction might act as a precursor to future studies. A reason not to construct GPS route buffers in the present study is the desire to be able to construct buffers that can be used for large population studies without having access to GPS data. Chaix et al. (6) describe how the strength of environment–behavior associations might decrease in GPS mobility studies compared to classical residential studies, indicating that the use of GPS to construct residential buffers suited for that particular behavior might prove useful.

One reason to keep the more traditional buffers is that they include and center around the residential address which focuses on the area close to home. Tobler’s first law of geography: “everything is related to everything else, but near things are more related than distant things” is very much linked to the argument that “overcoming space requires expenditure of energy and resources, something that nature and humans try to minimize” (33). That said, a tradeoff between area size and captured behavior is present and while smaller areas cannot capture all behavior, analyses within large areas includes built environments people never visit. One potential problem with buffer types that capture more of the daily cycling transport is that the significance of the closest neighborhood is diluted. By using city center-directed ellipse-shaped buffers, the residential address is kept as one of two important focus points, the other being a cluster of daily destinations. In doing so, the importance of the nearest neighborhood is acknowledged, yet the presumed area visited is kept relatively small. One could argue that work place is an important destination as well and that identifying an ellipse-shaped buffer based on the home–work route would also be useful, as well as a home–work–city center triangulation. This is speculative but nonetheless important to consider in future studies.

Findings of this study are not conclusive, but it seems that people living outside the city center generally cycle toward the city center (a cluster of destinations), with individual variations. Probably other factors such as age, education, gender, and income also affect the buffer (5). This suggests that future studies could benefit from using GPS to visualize movement patterns and construct more accurate representation of neighborhoods across population groups (children, elderly, pedestrians, cyclists, etc.). The use of GPS technology provides accurate representations of human–environment interactions in relation to, e.g., active transport and makes it possible to develop appropriate buffers (10).

The present results enable us to carry out analyses in a larger population using the city center-directed ellipse-shaped buffers studying the relationship between the built environment and transport-related physical activity (cycling and walking). The hypothesis for future studies is that the new buffers will better encapsulate transport cycling behavior and that the environmental characteristics in such buffers will show better correlation with behavior than the previously used buffers.

### Strengths and weaknesses

As the participants were recruited via the Danish IPEN study, several covariates had been collected via questionnaire, but for the present study only background data was analyzed and reported. The IPEN participants who participated in the study were regular cyclists which could have diminished the representativeness of the sample. However, a more random selection of participants could have resulted in large part of the participants not engaging in cycling for transport. The participants are however representative as cyclists, and as we wanted to study transport cycling behavior, this was crucial.

Even though GPS measures of transport behavior have proven beneficial in describing the actual movement, the use of GPS is still developing and is associated with challenges in both data collection and processing. Using GPS makes it possible to know the exact spatial footprint and measures of actual contact with the environment, but more often only potential contact is available. In this study, as in other studies using GPS, we had to overcome the typical problems with slow connectivity, satellite inference caused by physical structures or normal atmospheric conditions, and compliance in general (30). Furthermore, data processing and cleaning proved to be challenging but we managed to overcome some of the traditional problems like determining mode of transportation and an abundance of static points. By instructing the participants to wear the GPS when cycling and excluding all the data points which were not part of a trip as detected by PALMS, we were able to diminish the above mentioned. That said, we cannot rule out the possibility of errors and likewise acknowledge that the elliptical buffers might not adequately mimic individual transport behavior when transferred to other studies.

## Conclusion

In conclusion, GPS technology and geographical information systems are appropriate tools to study active transport behavior and subsequently display and analyze different buffer types, shapes, and sizes that best fit the behavior in question.

We found that transport patterns were affected by the distance from residential address to a cluster of destinations and that an elliptical-shaped buffer was more effective than traditional buffers such as network buffers or shortest route buffers in order to capture transport cycling in a Danish context. This has implications in studies of the relationship between the built environment and transport cycling. Acknowledging that constructing GPS-based individual buffers is not possible in most larger studies, we suggest using an elliptical buffer based on the distance and direction from home to a cluster of daily destinations (in Denmark often the city center) resulting in more circular buffers proximal to destinations and more elongated buffers for people living further from the cluster of destinations. The same approach might be advantageous in correlation with walking or other transport modes, as it seems plausible that most people move in direction of meaningful destinations. Meaningful destinations can vary from urban green spaces to shops, schools, or sport facilities so the buffer direction should reflect the study question and scope of the study.

## Author Contributions

Thomas Madsen and Jasper Schipperijn designed research, the conception of the study, and carried out the GPS and GIS work as well as data analysis. Thomas Madsen drafted the initial manuscript and revised it according to input from co-authors. Lars Breum Christiansen provided respondents and data from the Danish IPEN study, gave advice and input on the data analysis, and critically reviewed the manuscript. Thomas Sick Nielsen gave advice and input on the data analysis and critically reviewed the manuscript. Jens Troelsen conceived the original idea and critically reviewed the manuscript. All authors have responsibility for the final content and approve the final manuscript.

## Conflict of Interest Statement

The authors declare that the research was conducted in the absence of any commercial or financial relationships that could be construed as a potential conflict of interest.

## Footnotes

^1^www.ipenproject.org

^2^www.bt747.org
